# Oxidative Stress in the Developing Brain: Effects of Postnatal Glucocorticoid Therapy and Antioxidants in the Rat

**DOI:** 10.1371/journal.pone.0021142

**Published:** 2011-06-15

**Authors:** Emily J. Camm, Deodata Tijsseling, Hans G. Richter, Alexandra Adler, Jeremy A. Hansell, Jan B. Derks, Christine M. Cross, Dino A. Giussani

**Affiliations:** 1 Department of Physiology, Development and Neuroscience, University of Cambridge, Cambridge, United Kingdom; 2 Department of Obstetrics, University Medical Center, Utrecht, The Netherlands; Hôpital Robert Debré, France

## Abstract

In premature infants, glucocorticoids ameliorate chronic lung disease, but have adverse effects on long-term neurological function. Glucocorticoid excess promotes free radical overproduction. We hypothesised that the adverse effects of postnatal glucocorticoid therapy on the developing brain are secondary to oxidative stress and that antioxidant treatment would diminish unwanted effects. Male rat pups received a clinically-relevant tapering course of dexamethasone (DEX; 0.5, 0.3, and 0.1 mg.kg^−1^.day^−1^), with or without antioxidant vitamins C and E (DEXCE; 200 mg.kg^−1^.day^−1^ and 100 mg.kg^−1^.day^−1^, respectively), on postnatal days 1–6 (P1–6). Controls received saline or saline with vitamins. At weaning, relative to controls, DEX decreased total brain volume (704.4±34.7 mm^3^ vs. 564.0±20.0 mm^3^), the soma volume of neurons in the CA1 (1172.6±30.4 µm^3^ vs. 1002.4±11.8 µm^3^) and in the dentate gyrus (525.9±27.2 µm^3^ vs. 421.5±24.6 µm^3^) of the hippocampus, and induced oxidative stress in the cortex (protein expression: heat shock protein 70 [Hsp70]: +68%; 4-hydroxynonenal [4-HNE]: +118% and nitrotyrosine [NT]: +20%). Dexamethasone in combination with vitamins resulted in improvements in total brain volume (637.5±43.1 mm^3^), and soma volume of neurons in the CA1 (1157.5±42.4 µm^3^) and the dentate gyrus (536.1±27.2 µm^3^). Hsp70 protein expression was unaltered in the cortex (+9%), however, 4-HNE (+95%) and NT (+24%) protein expression remained upregulated. Treatment of neonates with vitamins alone induced oxidative stress in the cortex (Hsp70: +67%; 4-HNE: +73%; NT: +22%) and in the hippocampus (NT: +35%). Combined glucocorticoid and antioxidant therapy in premature infants may be safer for the developing brain than glucocorticoids alone in the treatment of chronic lung disease. However, antioxidant therapy in healthy offspring is not recommended.

## Introduction

Glucocorticoids are widely used to treat or prevent chronic lung disease (CLD) in preterm infants [1]–[5]. A major contributor to the development of CLD is excessive inflammation and thus glucocorticoids are used to prevent or reduce the severity of this complication due to their anti-inflammatory properties [4], [6], [7]. Despite established benefits, randomized clinical trials of postnatal steroid therapy have raised concerns regarding an increase in the rates of cerebral palsy and adverse neuromotor and cognitive outcomes [8], [9]. Furthermore, experimental studies in neonatal animals have demonstrated adverse effects of potent glucocorticoids, such as dexamethasone on brain growth, cell division, differentiation, myelination, apoptosis, and neurogenesis [10]–[15]. Clinically, therefore, the weaker glucocorticoid hydrocortisone is the steroid of choice [16]. The use of dexamethasone being particularly reserved for extremely premature (<32 weeks) and low birth weight infants unlikely to survive without therapy [17]. However, whilst hydrocortisone appears to cause fewer unwanted adverse effects than dexamethasone, it is also less potent in promoting the beneficial effects of glucocorticoids [16], [18], [19]. For these reasons, there is increasing clinical and basic science interest in persisting with the clinical use of potent steroids, such as dexamethasone, as long as the adverse side effects can be controlled. However, the mechanism underlying the unwanted side effects remains unknown, preventing the identification of plausible modified therapies to maintain the beneficial effects but prevent adverse side-effects.

Accumulating evidence suggests that glucocorticoids can promote oxidative stress in various tissues including the heart and vasculature [20]–[24] and kidney [21]. In relation to the brain, glucocorticoids increase the presence of reactive oxygen species (ROS) in both hippocampal and cortical cultures [25], and lower basal activity of cerebral antioxidant enzymes [26]. Furthermore, *in vitro* studies have shown that exposure to glucocorticoids can increase the susceptibility of cerebellar granule cells [27] and hippocampal neurons [28] to oxidative stress-induced cell death. Reactive oxygen species are produced under physiological conditions during metabolic reactions and functioning of the central nervous system [29]. However, excess ROS generation can cause cellular damage directly by attacking proteins, and indirectly, by generating further reactive species and initiating radical chain reactions [30]. Non-enzymatic and enzymatic antioxidants provide defence mechanisms against excess ROS production. The former include the water soluble vitamin C and the fat soluble vitamin E. Vitamin C is the most effective aqueous-phase antioxidant in human blood plasma because of its ability to trap peroxyl radicals, thereby preventing lipid peroxidation [31]. Vitamin E is a peroxyl radical scavenger and one of the most important inhibitors of the free-radical chain reaction of lipid peroxidation, giving it a major role in protecting biological membranes [32]. Antioxidant treatment has been shown to prevent and partially restore glucocorticoid-induced vascular dysfunction [23] and hypertension [24], further supporting the premise that oxidative stress may be the underlying mechanism mediating unwanted side-effects of glucocorticoids.

Whilst ROS have been implicated in the pathogenesis of various neurodegenerative disorders including Parkinson's and Alzheimer's disease [33]–[35], the role of ROS in the pathogenesis of glucocortocoid-induced brain injury during early development, remains unclear. In this study, we have combined these separate lines of evidence to propose the inter-related hypotheses that the adverse effects of postnatal glucocorticoid therapy on the developing brain are secondary to oxidative stress and that antioxidant treatment would diminish unwanted effects. The hypothesis was tested using a well established model of postnatal glucorticoid therapy in rats [36], [37].

## Methods

### Ethical Approval

The study was approved by the Cambridge University Ethical Review Committee. All procedures were carried out under the UK Animals (Scientific Procedures) 1986 Act and conducted under the authority of the appropriate project licence.

### Animals and Experimental Design

Twenty-five pregnant Wistar rats (Charles River, UK) with timed gestations were individually housed under standard conditions (23±1°C, light∶dark, 12∶12 hour) with access to food (Special Diet Services, UK) and water. All dams delivered on day 22 of gestation (assigned postnatal day 0, P0). Litters were then divided into four treatment groups: control (Ctrl, n = 6), dexamethasone (DEX, n = 6), dexamethasone with vitamins C and E (DEXCE, n = 7), and control with vitamins C and E (CtrlCE, n = 6).

Within 3–5 hours of birth, pups were sexed and weighed, and litters reduced to 8 pups per dam (four males and four females) in order to standardize postnatal nutrition and maternal care. To account for sex differences, only male pups within each litter received treatment (Ctrl, n = 24; DEX, n = 24; DEXCE, n = 28; and CtrlCE, n = 24). Male pups received two intraperitoneal (i.p.) injections daily (10 µL.g^−1^ for each) of some or all of the following solutions from Sigma (Sigma-Aldrich, UK): Dexamethasone (Dexamethasone-21-phosphate, disodium salt), vitamin C (L-ascorbic acid), and vitamin E (*dl*-α-tocopherol acetate). Two injections were used due to the different solubilities of vitamin E (dissolved in groundnut oil) and vitamin C and dexamethasone (both dissolved in 0.9% NaCl). Ctrl pups received injections of saline and groundnut oil for the duration of the treatment period, postnatal days 1–6 (P1–6). Dexamethasone pups received a three-day, tapering course of dexamethasone (0.5, 0.3, and 0.1 mg.kg^−1^.day^−1^) plus separate injections of oil on P1–P3 and then only saline and oil from P4–P6. DEXCE pups received the same treatment as DEX pups, except that vitamins C (200 mg.kg^−1^.day^−1^) and E (100 mg.kg^−1^.day^−1^) were administered over the entire treatment period in addition to the three-day tapering course of dexamethasone. CtrlCE pups received injections of vitamin C and E from P1–6. The dose and duration of dexamethasone used in this study was derived from and is proportional to the 21-day tapering course of dexamethasone used in human preterm infants to prevent or lessen CLD, starting at 0.5 mg.kg^−1^ [36]. Ascorbic acid and α-tocopherol are often combined to promote antioxidant activity; this combination is essential for preventing pro-oxidant activity of the α-tocopheroxyl radical, which is formed from α-tocopherol during lipid peroxidation [32], [38]. Ascorbate regenerates α-tocopherol from α-tocopheroxyl radicals, thus preserving the antioxidant role of α-tocopherol [38], [39]. The doses of vitamins C and E used in this study were adopted from studies indicating successful antioxidant effects in adult Wistar rats at these levels [40]. Body weight (BW) from P0 to P7 and every other day thereafter was recorded.

### Tissue collection

During the treatment period, 10 male DEX pups, 2 male DEXCE pups and 1 male Ctrl pup died, leaving the following remaining male pups for study (Ctrl, n = 23; DEX, n = 14; DEXCE, n = 26; and CtrlCE, n = 24). On P21, approximately half of these surviving male pups from each litter (Ctrl: n = 11; DEX: n = 7; DEXCE: n = 12; CtrlCE: n = 12) were deeply anaesthetised (0.2 mL total volume, i.p., 100 mg.mL^−1^ ketamine, Fort Dodge Animal Health, UK and 20 mg.mL^−1^ xylazine, Millpledge Veterinary, UK). To assess the symmetry of growth, BW and crown-rump length (CRL) were determined. Brain tissue was weighed and dissected. The hippocampal formation was separated from the overlying cortex using a dissecting microscope [41], then snap frozen in liquid nitrogen and stored at −80°C for subsequent Western blot analysis. On P22, the remaining male pups (Ctrl: n = 12; DEX: n = 7; DEXCE: n = 14; CtrlCE: n = 12) were deeply anaesthetised as described above and perfused intracardially with a NaCl solution (10 mM PIPES, 139 mM NaCl, 2.7 mM KCl, 19.4 mM D-glucose, 7.5 µM PVP, pH 7.2) followed by 4% paraformaldehyde (PFA). Brains were collected, weighed and stored overnight at 4°C in 4% PFA for subsequent stereological analysis. Dissection for isolation of tissue for freezing and fixing was performed on separate days to accommodate the number of animals to be processed within a 24-hour period.

### Histology and stereology

#### Tissue preparation

The cerebrum was exhaustively sectioned at 50 µm using a Leica RM 2235 vibratome (Leica Microsystems, Germany). Tissue was then stored in cyroprotectant at −20°C until histological and immunohistochemical staining was performed. All subsequent quantitative analyses were performed with the observer blind to the treatment group.

#### Immunohistochemistry

Sections were washed for 30 minutes in phosphate buffered saline (PBS, Sigma-Aldrich, U.K.), incubated with 30% H_2_O_2_ for 5 min to block endogenous peroxidase activity and washed in PBS for 30 minutes. Subsequently non-specific binding was blocked with 4% BSA in PBS for 10 min. Sections were then incubated with primary antibody (MBP; 1∶400, Millipore, UK; NeuN, 1∶400, Millipore, UK) in primary diluent (2% BSA in PBS containing 0.3% Triton; Sigma Aldrich, U.K.) overnight. The following day sections were washed 30 minutes in PBS, incubated for 1 hour with secondary antibody (1∶400, Vector Laboratories, USA) in secondary diluent (2% BSA in PBS) then washed for 15 minutes in PBS. Sections were incubated for 1 hour in AB (Vector Laboratories, U.K.) in PBS then washed in PBS for 15 minutes. Staining was visualized by adding metal DAB (Thermo Scientific, UK) in peroxide buffer (Thermo Scientific, UK) for 2 minutes to the sections. Tissue was then mounted with 0.5% gelatine in PBS on slides.

#### Volumetric analysis

To assess the volume of the cerebrum and its compartments, systematic random sampling [42] was used to select, without bias, 10 sections per animal for analysis. Selected sections were stained using 1% Cresyl Violet. A point grid was superimposed on the sections and viewed using a ×1.25 objective. The volume of both the left and right hemispheres, the cortex, white matter, and the hippocampus, were analysed. Points falling on each compartment were counted and the Cavalieri principle (Gundersen et al, 1998) was applied in order to calculate estimated volumes:
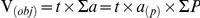
Where V*_(obj)_* represents the estimated volume of the brain region, t is the total length of the brain (*t* = no. of sections×section thickness), *a*_(p)_ is the area associated with each point and Σ*P* is the sum of points for that formation. All volumetric quantifications were performed with an Olympus BX-50 microscope and CAST grid.

#### Neuronal number and soma volume quantification

**Figure 1:**
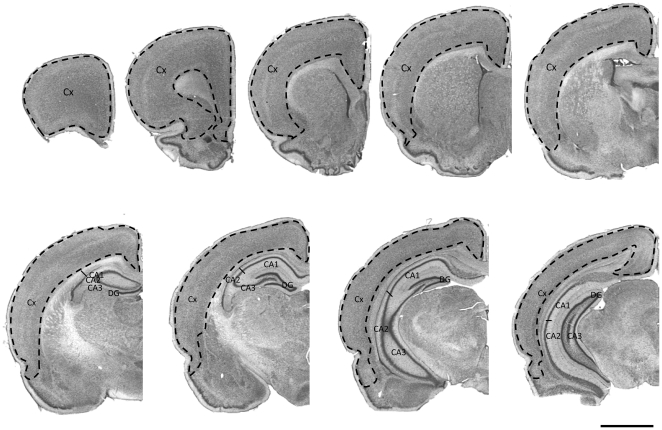
Photomicrograph of coronal brain section. Cresyl violet-stained sections showing the regions used for determining neuronal number and soma volumes. These parameters were measured in the following fields: cortex (Cx), CA1, CA2/3 and dentate gyrus (DG). Scale bar = 2.5 mm.

Neuronal number was estimated by using the fractionator method. All available NeuN stained sections containing the cortex and hippocampus (divided into the CA1, CA2/3 and dentate gyrus) were sampled using an Olympus BX-50 microscope and CAST grid (Fig. 1). Step motors on the microscope were used to randomly sample a known fraction of the tissue. This was achieved by calibrating the lengths of the X and Y steps. An unbiased counting frame of known dimensions was superimposed on the tissue image. Nuclei within the counting frame, or those touching the permitted lines of the counting frame, were counted. To calculate total neuronal number, the following formula was applied:
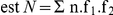
where est *N* is the estimated total number of nuclei, Σ n is the sum of nuclei counted in the brain sample, f_1_ is the reciprocal of the sampling fraction, and f_2_ is the areal sampling fraction.

The soma volume of neurons from the cortex and the different regions of the hippocampus were calculated using the vertical dissector tool in the CAST grid programme, which generates two orthogonal lines through the mid-point of the neuron. The boundaries of the neuron were then identified at the intersections of these lines with the cell membrane. Soma volume was calculated using the following formula:
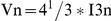
where In refers to the distance from the mid-point of the neuron to the cell membrane of the neuron.

#### Myelination

To assess the extent of myelination, the optical density (OD) of MBP-stained fibres was measured in the corpus callosum and cortex using ImageJ (V1.38, National Institutes of Health). On the basis of anatomical landmarks, equivalent sections from all rats were chosen. A calibrated optical density step tablet was used to calibrate the images to optical density. Eight fields within the corpus callosum and cortex were examined over three sections per animal. Optical density was measured as grey levels. Non-specific background ODs were measured at each brain level in a region devoid of MBP-immunostaining and these were subtracted from the corpus callosum and cortex values. The area of MBP-positive fibres in the cortex, relative to cortical size, was also assessed using the same software.

### Western blotting

Western blots were performed using 20 µg aliquots of protein, resolved on 10–12% SDS-PAGE gels. Proteins were transferred to polyvinylidene fluoride Immobilon-P membranes (Millipore, UK) by electroblotting. Membranes were blocked at room temperature for 1 hour with 5% dry skim milk in tris-buffered-saline containing 1% Tween-20 (TBS-T, Sigma-Aldrich, UK). Purified antibodies to β-actin (1∶50,000, Sigma-Aldrich, UK), nitrotyrosine (NT; 1∶2,000, Zymed Laboratories, USA), 4-hydroxynonenal (4-HNE; 1∶2,000, Calbiochem, Germany), or heat shock protein 70 (Hsp70; 1∶20,000, Stressgen, UK) in 5% milk in TBS-T were added, and incubated at 4°C overnight. Membranes were washed in TBS-T, incubated for 1 hour in a secondary antibody conjugated to horseradish peroxidase (donkey anti-rabbit IgG or sheep anti-mouse IgG; 1∶10,000, GE Healthcare, UK) and washed in TBS-T. Proteins were visualised using enhanced chemiluminescence (ECL, Amersham, UK), exposed to X-ray film and films were developed (Fuji FPM100A Processor). Bands densities were quantified and expressed relative to β-actin (ImageJ software, NIH).

### Statistical Analysis

Data are presented as mean ± SEM unless otherwise indicated. Data were analysed by One-Way ANOVA followed by the Student-Newman-Keuls or Tukey *post hoc* test. SigmaStat software (SigmaStat for Windows, Version 2.0) was used for all statistical analyses. P values<0.05 were accepted as statistically significant.

## Results

### Body and brain weights

**Figure 2:**
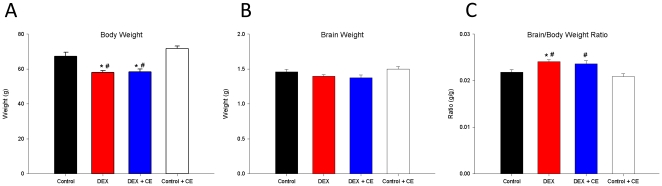
Body, brain and brain: body weight. (A) Body weight, (B) brain weight and (C) brain: body weight at postnatal day 22 in control (Ctrl, n = 5), dexamethasone (DEX, n = 5), dexamethasone with vitamins C and E (DEXCE, n = 5), and control with vitamins C and E (CtrlCE, n = 5) pups. *, P<0.05 *versus* Ctrl; #, P<0.05 *versus* CtrlCE (One-Way ANOVA+Student-Newman-Keuls).

Compared to control offspring, DEX, with or without vitamins, reduced body weight at weaning (Fig. 2A; P<0.05) Absolute brain weight was not altered between the groups (Fig. 2B; P>0.05), however, the brain to body weight ratio was significantly increased in DEX offspring, but not DEXCE offspring when compared to Ctrl offspring (Fig. 2C; P<0.05).

### Histological Analysis

#### Gross morphology

Examination of the cresyl violet-stained coronal sections did not reveal any gross alterations in cytoarchitecture, cellular morphology, or anatomical organisation of the cortex or hippocampus between the groups at P22.

#### Regional brain volumes

**Figure 3:**
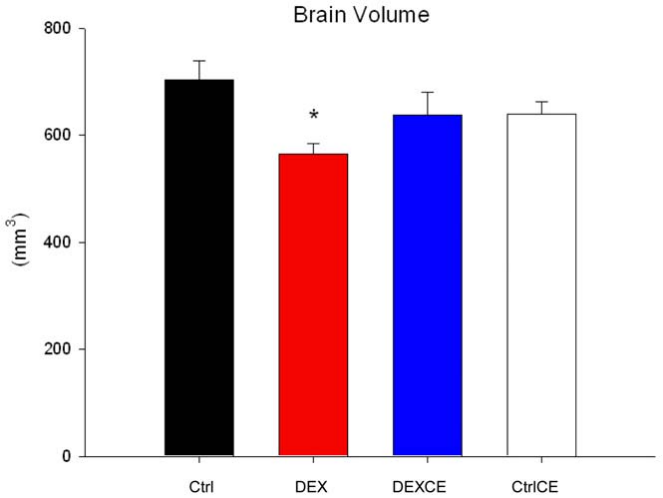
Cerebrum brain volume. Brain volume of the cerebrum at postnatal day 22 in control (Ctrl, n = 5), dexamethasone (DEX, n = 5), dexamethasone with vitamins C and E (DEXCE, n = 5), and control with vitamins C and E (CtrlCE, n = 5) pups. *, P<0.05 *versus* Ctrl (One-Way ANOVA+Student-Newman-Keuls).

cytoarchitectureThe volume of the cerebrum was significantly decreased in DEX offspring (Fig. 3; P<0.05). Dexamethasone in combination with vitamins did not significantly alter total brain volume (P<0.05). No further alterations in absolute regional volumes were observed between the groups (data not shown; P>0.05). Absolute regional volumes and regional volumes relative to total brain volume, were also unaltered (data not shown; P>0.05).

#### Neuronal numbers and soma volumes

**Figure 4:**
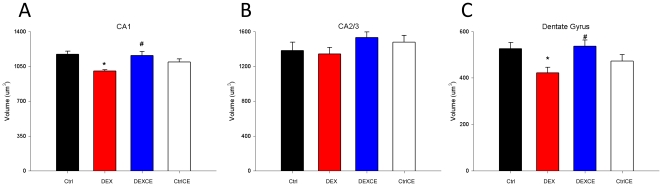
Soma volumes in the CA1 (A), CA2/3 (B), and dentate gyrus (C) of the hippocampus. Soma volumes in the hippocampus are shown from the control (Ctrl, n = 5), dexamethasone (DEX, n = 5), dexamethasone with vitamins C and E (DEXCE, n = 5), and control with vitamins C and E (CtrlCE, n = 5) groups. *, P<0.05 *versus Ctrl*; #, P<0.05 *versus* DEX (One-Way ANOVA+Student-Newman-Keuls).

There were no differences between the groups in neuronal numbers in either the cortex or hippocampus (Table 1, P>0.05). However, in DEX-treated offspring compared to control offspring, soma volume was significantly reduced in both the CA1 region and the dentate gyrus of the hippocampus (Fig. 4 A, C; P<0.05). Soma volume was not different between the groups in the CA2/3 region of the hippocampus (Fig. 4B; P>0.05). Soma volumes in the CA1 and dentate gyrus were unaltered in DEXCE offspring (P<0.05).

**Table 1:** Neuronal numbers in the cortex and hippocampus.

	Ctrl	DEX	DEXCE	CtrlCE
Neuronal Number in Cortex (million)	20.4±1.1	19.0±0.6	18.3±0.6	20.9±10.7
Cortical Reference Volume (mm^3^)	343.0±14.4	293.8±9.6	335.8±26.6	321.0±16.7
Neuronal Number in CA1 (×1000)	410.1±24.5	413.8±17.7	464.4±28.4	452.6±14.8
CA1 Reference Volume (mm^3^)	0.79±0.01	0.81±0.07	0.86±0.07	0.92±0.07
Neuronal Number in CA2/3 (×1000)	440.8±28.2	438.6±34.2	400.8±23.7	416.3±33.1
CA2/3 Reference Volume (mm^3^)	0.73±0.10	0.81±0.02	0.75±0.05	0.81±0.10
Neuronal Number in Dentate Gyrus (×1000)	870.0±69.1	932.5±47.7	887.2±54.1	868.9±94.7
Dentate Gyrus Reference Volume (mm^3^)	0.64±0.04	0.69±0.04	0.76±0.04	0.78±0.08

Neuronal numbers and reference volumes in the control (Ctrl, n = 5), dexamethasone (DEX, n = 5), dexamethasone with vitamins C and E (DEXCE, n = 5) and control with vitamins C and E (CtrlCE, n = 5) pups. The reference volumes are the volumes of the cortex and compartments of the hippocampal formation (CA1, CA2/3 and dentate gyrus) that neuronal number and soma volumes were measured in.

#### Myelination

**Figure 5:**
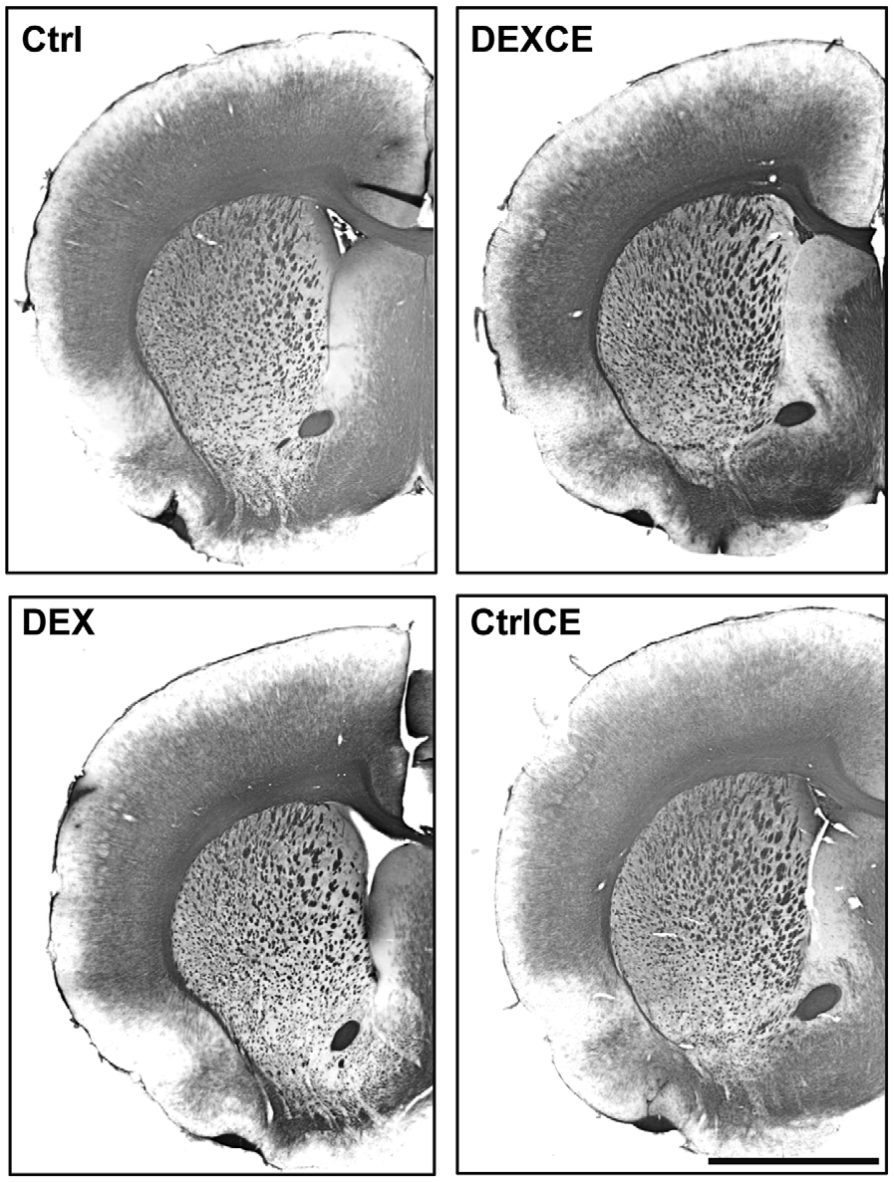
Photomicrograph of myelin staining. Coronal section of control (Ctrl), dexamethasone (DEX), dexamethasone with vitamins C and E (DEXCE), and control with vitamins C and E (CtrlCE)-treated offspring stained with myelin basic protein (MBP). Overall, the optical density (OD) of myelin staining in the corpus callosum was significantly increased in DEXCE offspring when compared to Ctrl offspring (P<0.05; One-Way ANOVA+Student-Newman-Keuls). Scale bar = 2.5 mm.

The OD of MBP-stained fibres was significantly increased in the corpus callosum of DEXCE offspring when compared to Ctrl offspring (Ctrl: 0.211±0.006; DEX: 0.251±0.029 DEXCE: 0.299±0.021; CtrlCE: 0.259±0.016, Fig. 5; P<0.05). There were no significant differences between the groups in relation to the OD of MBP-stained fibres in the cortex (Ctrl: 0.184±0.008; DEX: 0.176±0.024; DEXCE: 0.204±0.014; CtrlCE: 0.185±0.01; P>0.05), or the extent of myelination (Ctrl: 58.9±0.8%; DEX: 58.9±0.8%; DEXCE: 56.5±1.0%; CtrlCE: 61.9±2.3%; P>0.05).

### Western blot analysis

**Figure 6:**
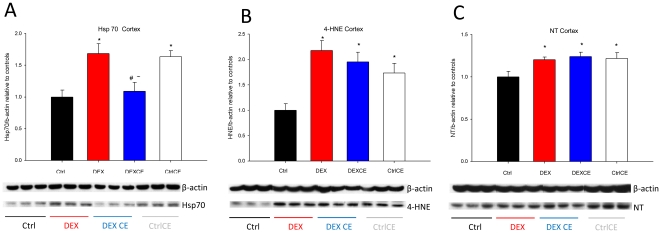
Expression of heat-shock protein 70 (Hsp70, 70 KDa, A), 4-hydroxynonenal (4-HNE, 54 kDa, B), and nitrotyrosine (NT, 35 kDa, C) in the cortex. Representative Western blots are shown from the control (Ctrl, n = 6), dexamethasone (DEX, n = 6), dexamethasone with vitamins C and E (DEX CE, n = 6), and control with vitamins C and E (CtrlCE, n = 6) groups. Expression of the proteins was quantified and expressed as a ratio to β-actin. *, P<0.05 *versus* Ctrl; #, P<0.05 *versus* DEX; ∼, P<0.05 *versus* CtrlCE (One-Way ANOVA+Student-Newman-Keuls).

At P21, DEX increased the protein expression in the cortex of three established molecular indices of oxidative stress: Hsp70 (+68%), 4-HNE (+118), and NT (+20%, Fig. 6A–C; P<0.05). Dexamethasone in combination with vitamins did not significantly alter Hsp70 protein expression relative to control or DEX-treated offspring (+9%; P<0.05). However, 4-HNE (+95%) and NT (+24%) remained up-regulated in the cortex (P<0.05). Relative to control offspring, no significant alterations in protein expression were observed in the hippocampus of DEX (Hsp70: +4%; 4-HNE: −6%; NT: −5%, data not shown; P>0.05) or DEXCE offspring (Hsp70: −21%; 4-HNE: −1% ; NT: −28%, data not shown; P>0.05). Treatment of neonates with vitamins alone increased Hsp70 (+63%) and 4-HNE (+73%) in the cortex (Fig. 6 A, B; P<0.05,), and NT in both the cortex (+22%, Fig. 6C; P<0.05) and hippocampus (+35%, data not shown; P<0.05).

## Discussion

The data show that postnatal treatment with dexamethasone in human clinically-relevant doses decreased total brain volume and reduced neuronal soma volume in the CA1 and dentate gyrus of the hippocampus. The study adds the novel observation that co-administration of antioxidant vitamins with dexamethasone improved total brain volume and neuronal soma volumes in the hippocampus. Postnatal treatment with dexamethasone also increased three molecular indices of oxidative stress in the cortex. Co-administration of antioxidant vitamins restored Hsp70 protein expression in the cortex, however the expression of 4-HNE and NT remained significantly elevated. Treatment of neonates with vitamins alone induced oxidative stress in the cortex.

In the present study, morphological analysis of brain tissue revealed that volume of the cerebrum was significantly decreased in dexamethasone-treated offspring when compared to controls. Importantly, co-administration of dexamethasone with antioxidant vitamins prevented this effect. As there were no regional alterations in brain volumes when expressed relative to total brain volume, it appears that the brains of the dexamethasone-treated offspring were proportionally smaller. A decrease in whole cerebral tissue volume may be explained by reductions in neuronal numbers, soma volumes and/or delayed dendritic growth. To explore these possibilities, total neuronal numbers and soma volumes were assessed in the cortex and hippocampus, respectively. Total neuronal number was not altered following dexamethasone treatment in either the cortex or hippocampus. However, in dexamethasone-treated offspring, soma volume was reduced in the CA1 and the dentate gyrus of the hippocampus. Smaller neuronal volumes may be related to a decline in cellular function [43], selective loss of larger neurons, which has been observed in neurodegenerative diseases [44], or reflect neuronal shrinkage, which often precedes neurodegeneration [45]. Dexamethasone may delay neuronal differentiation or dendritic growth [13], which, in addition to the reduction in soma volumes, may also account for the reduction in brain volume. Antioxidant vitamin supplementation in dexamethasone-treated offspring prevented the decrease in hippocampal soma volumes. The data provide evidence for the powerful neuroprotective effects of vitamins C and E following glucocorticoid therapy.

To explore the mechanisms via which clinically-relevant doses of dexamethasone affected cerebral volume and the beneficial effects of vitamins C and E, the present study focussed on three established molecular indices of oxidative stress known to be affected by glucocorticoid treatment. In humans, corticosteroid therapy has been reported to markedly increase levels of 4-HNE [46] and NT immunoreactivity [23]. In addition, it is established that dexamethasone decreases eNOS protein and/or mRNA expression in several tissues in the rat [20], [47], [48]. Dexamethasone decreases eNOS activity, largely by limiting substrate availability, thereby promoting eNOS uncoupling leading to further superoxide generation and reduced NO bioavailability [20], [22], [47], [49]. In the present study, dexamethasone increased the expression of Hsp70, 4-HNE, and NT in the cortex. Vitamin administration attenuated the increase in Hsp70 protein expression induced by dexamethasone treatment; however, the up-regulation of 4-HNE and NT remained unchanged. Similarly, previous studies have reported that vitamin C supplementation attenuated the increase in Hsp70 expression in muscle following physiological oxidative stress induced by exercise [50]. Vitamin C and E supplementation in combination could also decrease Hsp70 expression in the brain following heat stress [51]. Data in the present study therefore confirm that clinically-relevant doses of dexamethsone induce oxidative stress in the developing brain and that antioxidant therapy partially protect against this effect. The lack of demonstrable structural and neuronal injury, particularly in the cortex where oxidative stress was evident, may not necessarily imply normal function following postnatal glucocorticoid therapy. For instance, oxidative or nitrosative stressed neurons, injured by free radicals, might be present physically but be non-functional [52], [53]. Further, clinical evidence does suggest that early postnatal dexamethasone therapy can have long-term adverse neuromotor and cognitive outcomes [8], [9].

The physiology underlying the inhibitory effects of antioxidant vitamins on the expression of Hsp70 in past and present studies is likely multi-factorial and these mechanisms may also relate to the protective effect of antioxidants against glucocorticoid-induced reduction in cerebral volume, as shown in the present study. For instance, in addition to quenching ROS directly [39], antioxidant vitamins can act through tetrahydrobiopterin (BH4) to stimulate NO production by endothelial NO synthase [54]. Antioxidant vitamins can also prevent the S-nitrosylation of sensitive thiol groups on endothelial NO synthase, thereby maintaining their stability and the affinity of NO synthase for BH4 [55]. Further, it has been reported that vitamin C may enhance the expression and/or action of potent antioxidant enzymes [56]. Since manipulation of the vascular NO∶^•^O_2_^−^ ratio is an important determinant of vascular tone, whereby a decrease in the ratio promotes vasoconstriction and an increase leads to vasodilatation [57], glucocorticoid-induced oxidative stress will favour a decrease in perfusion and antioxidant therapy will oppose this. Accordingly, several studies have shown that clinically-relevant dosing regimens of glucocorticoids in perinatal practice increase vascular resistance in several vascular beds, including the cerebral circulation [58]–[60]. Any effects of antioxidant vitamins which enhance the bioavailability of NO will favour maintained perfusion and thereby a protection against the stunting effects of glucocorticoids on growth, particularly on circulations which are highly dependent of NO, such as the cerebral vascular bed.

The mechanisms underlying the region-specific pattern of cerebral oxidative stress reported in the present paper are not well understood. One explanation may be that the neuronal populations in the cortex show greater vulnerability to oxidative stress as a result of a greater oxidative burden and/or lower antioxidant protection. Alternatively, it is possible that the hippocampus may have shown early changes in markers of oxidative stress following postnatal glucocorticoid treatment, but that these indices were below the threshold of detection with available techniques or that they had subsided by the time of tissue collection.

Additional data in the present study show that treatment of neonates with vitamins alone increased Hsp70 and 4-HNE in the cortex, and NT in both the cortex and hippocampus. Although antioxidant vitamin supplementation may improve disease states and/or conditions associated with enhanced oxidative stress, antioxidant treatment in healthy conditions where the physiology of the individual is already replenished with an appropriate equilibrium of pro- and anti-oxidant mechanisms, may, in fact, lead to excess NO bioavailability. The latter will promote peroxynitrite generation, thereby mimicking oxidative stress with subsequent induction of a number of the unwanted side-effects [61], [62].

Postnatal dexamethasone treatment, with or without vitamins, significantly reduced body weight at P22. Maternal antenatal treatment with glucocorticoids has been shown to reduce weight gain in human infants [63] and suppress growth until weaning in rats [64]. Postnatally, glucocorticoids reduce somatic growth in premature human infants [65]. Our results also confirm similar findings reported in other experimental studies [36], [37], [66]–[68]. This reduction in somatic weight is likely due to the well-described direct effects of glucocorticoids on overall tissue accretion and catabolism [69]–[71] rather than the effects of glucocorticoid-induced oxidative stress on perfusion of regional circulations. Dexamethasone appeared to have a greater effect on body weight than brain weight (brain sparing), which suggests an increase in vascular resistance in peripheral circulations. The peripheral vasoconstrictor effects of dexamethasone in early life are well known. Two separate studies have reported that treatment of fetal sheep in late gestation with glucocorticoids leads to an increase in vascular resistance in peripheral circulations [58], [59]. In this study, dexamethasone treatment reduced survival to 70%, a finding supported by other studies in neonatal rat pups [72]–[74]. This increase in mortality may be attributed to an inability of the offspring to utilise nutrients, which may be due to decreased suckling and/or accelerated gut closure [75].

In conclusion, postnatal glucocorticoid treatment in human clinically relevant doses reduced total brain volume and soma volumes in the hippocampus and induced oxidative stress in the developing brain. Combined glucocorticoid and antioxidant treatment may ameliorate some of the adverse consequences of postnatal glucocorticoid therapy on the developing brain. However, antioxidant vitamin supplementation is not recommended in healthy offspring. The data provide the proof of concept that antioxidant therapy may antagonise some of the unwanted effects of potent glucocorticoids needed in clinical practice. It is now important to investigate the value of this combined therapy on cerebral structure and functional development in an experimental model that closely mimics conditions associated with premature birth in humans, including established chronic lung disease or bronchopulmonary dysplasia.
